# Adipokine Hormones and Hand Osteoarthritis: Radiographic Severity and Pain

**DOI:** 10.1371/journal.pone.0047860

**Published:** 2012-10-26

**Authors:** Mei Massengale, Bing Lu, John J. Pan, Jeffrey N. Katz, Daniel H. Solomon

**Affiliations:** 1 Division of Rheumatology, Immunology, and Allergy, Brigham and Women's Hospital, Boston, Massachusetts, United States of America; 2 Division of Pharmacoepidemiology and Pharmacoeconomics, Brigham and Women's Hospital/Harvard Medical School, Boston, Massachusetts, United States of America; 3 Department of Orthopedic Surgery, Brigham and Women's Hospital, Boston, Massachusetts, United States of America; 4 Orthopaedic and Arthritis Center for Outcomes Research, Brigham and Women's Hospital, Boston, Massachusetts, United States of America; 5 Department of Epidemiology, Harvard School of Public Health, Boston, Massachusetts, United States of America; 6 Harvard Medical School, Boston, Massachusetts, United States of America; 7 Division of Musculoskeletal Radiology, Department of Radiology, Brigham and Women's Hospital, Boston, Massachusetts, United States of America; 8 Center for Regenerative Medicine, Massachusetts General Hospital, Boston, Massachusetts, United States of America; University of Texas Health Science Center at San Antonio, United States of America

## Abstract

**Introduction:**

Obesity's association with hand osteoarthritis cannot be fully explained by mechanical loading. We examined the relationship between adipokines and radiographic hand osteoarthritis severity and pain.

**Methods:**

In a pilot study of 44 hand osteoarthritis patients (39 women and 5 men), serum adipokine concentrations and hand x-ray Kallman-scores were analyzed using linear regression models. Secondary analyses examined correlates of hand pain.

**Results:**

The cohort had a mean age of 63.5 years for women and 72.6 for men; mean (standard deviation) Kallman-scores were 43.3(17.4) for women and 46.2(10.8) for men. Mean body-mass-index was 30 kg/m^2^ for women and men. Mean leptin concentration was 32.2 ng/ml (women) and 18.5 ng/ml (men); mean adiponectin-total was 7.9 ng/ml (women) and 5.3 ng/ml (men); mean resistin was 7.3 ng/ml (women) and 9.4 ng/ml (men). No association was found between Kallman-scores and adipokine concentrations (R^2^ = 0.00–0.04 unadjusted analysis, all p-values>0.22). Secondary analyses showed mean visual-analog-scale pain of 4.8(2.4) for women and 6.6(0.9) for men. Leptin, BMI, and history of coronary artery disease were found to be associated with visual-analog-scale scores for chronic hand pain (R^2^ = 0.36 unadjusted analysis, p-values≤0.04).

**Conclusion:**

In this pilot study, we found that adipokine serum concentrations were not associated with hand osteoarthritis radiographic severity; the most important correlates of joint damage were age and disease duration. Leptin serum concentration, BMI, and coronary artery disease were associated with the intensity of chronic hand OA pain.

## Introduction

Osteoarthritis (OA) is the most common joint disease worldwide and can affect any joint [1]. OA-related disability and joint surgeries pose high costs to individuals and society [1]. Obesity is a known risk factor, presumably due to excessive joint loading [2]. Intriguingly, studies also suggest an association between obesity and osteoarthritis of the hand, a non-weight bearing joint [2]. The mechanism underpinning this association, however, is not clear.

Studies have linked the adipocyte-secreted adipokine hormones (leptin, adiponectin, and resistin) with diseases of cartilage, such as OA [3]. We chose to examine hand OA and its potential association with adipokines as a model to minimize the confounding effect of mechanical loading on joints due to weight-bearing. We recruited a cohort of 44 hand OA patients and examined the cross-sectional relationship of adipokine serum concentrations with hand OA radiographic severity and pain.

## Patients and Methods

### Study Population

Eligible participants were identified through a centralized clinical data registry, the Partners Research Patient Data Registry (RPDR). We selected subjects from this population using the following inclusion criteria: hand OA [4], age>45 years, availability of at least one hand x-ray for review, and at least one outpatient visit at our hospital since January 1, 2008. Exclusion criteria included inflammatory or active crystalline arthritis, intra-articular fracture history, diagnosis of significant medical comorbidities, or pregnancy.

### Recruitment Method

Per study protocol approved by Partners Health Care Institutional Review Board, we sent invitation letters to eligible patients. The first author (MM) made follow-up phone calls with preliminary screening. Eligible subjects were invited for the study visit. After giving written consent, subjects received an interview, a hand-examination, and phlebotomy. All samples were collected in mornings after overnight fast. This work was approved by Brigham and Women's Partners Institutional Review Board.

### Data Elements

The study outcome was the Kallman hand OA x-ray score [5]. The most recent hand x-ray was graded by a rheumatologist (MM) trained by a board-certified musculoskeletal radiologist (JP), using Kallman scoring system on a scale of 0–99 [5]–[7]. If bilateral hand x-rays were available, the worse side was selected for assessment. Ten randomly selected hand x-rays were scored by MM, at two separate occasions a few weeks apart, with prior scores blinded. The intra-rater reliability estimated by the intra-class correlation coefficient was 0.78. These 10 hand x-rays were also graded by musculoskeletal radiologist (JP) with prior scores blinded. Inter-rater reliability was estimated by intra-class correlation of 0.71. At the visit, subjects reported the intensity of chronic hand pain on a visual-analog-scale (VAS), as a response to the question: “On an average day, what is the typical score for your hand pain?” [8]. Confounders assessed were age, gender, race, body-mass-index, family history of OA, history of polyarticular OA, history of coronary artery disease (CAD), history of thyroid disease, diabetes mellitus (DM), and hand OA disease duration estimated from the time of initial onset of hand pain.

### Biochemical Assays

All specimens were processed in Harvard Catalyst Central Laboratory, 221 Longwood Avenue, Boston, MA 02115. Serum leptin Radioimmunological Assay Kit brand was Millipore, St. Charles, Missouri; adiponectin multimeric enzyme-linked immunosorbent assay (ELISA) Kit brand was ALPCO Diagnostics Inc, Salem, NH; and resistin Quantikine ELISA Kit brand was R&D Systems, Minneapolis, MN. The Intra-assay variation and inter-assay variation were 5.2–7.5% and 3.2–8.9% for leptin; 5.0–5.4% and ∼6% for adiponectin total; 3.3–5.0% and ∼6% for adiponectin high molecular weight fraction; 10.2–13.7% and ∼14% for adiponectin middle molecular weight fraction; and 1.8–7.7% and 3.4–9.3% or resistin.

### Statistical Analysis

The primary analyses examined the relationship between Kallman scores and adipokine concentrations. To perform secondary analyses on the relationship of hand pain VAS scores with adipokines, we excluded subjects with co-morbidities potentially contributing to hand pain, such as fibromyalgia, hepatitis C, and carpel tunnel syndrome.

Both unadjusted and multivariable adjusted linear regression models were performed. We also performed adjusted analyses of all covariates without forcing in adipokine serum concentrations. The validity of model assumptions was assessed using analysis of residuals. Statistical significance was assessed by examining p-values at a two-sided significance level of 0.05 unless otherwise stated. We used SAS 9.3 (SAS Institute, Inc, Cary, North Carolina) for all analysis.

## Results

We reviewed 2316 medical charts from the RPDR, of which 376 were found to meet the study criteria. Primary rheumatologists approved 210 patients for investigators to contact. Forty-five (21% of women and 25% of men contacted) consented to the study. For patients who did not consent to the study, the average age was 62 for women and 69 for men, similar to that of the participants (Table 1). The common reasons for refusal were inconvenience, transportation difficulties, weather, health problems unrelated to hand OA, and inability to fast overnight.

**Table 1:** Demographics, Disease Characteristics, and Adipokine Serum Concentrations of Study Participants Categorized by Gender.

	Female	Male
	N = 39	N = 5
	Mean ± SD or N (%)	
Age	63.5±8.3	72.6±8.4
Age		
45–50	2 (5.1)	0
51–60	11 (28.2)	0
61–70	14 (35.9)	2 (40.0)
71–80	12 (30.8%)	2 (40.0)
80+	0	1 (20.0)
BMI (kg/m^2^)	30.0±7.3	29.5±4.7
BMI status		
Normal (BMI<25)	9 (23.1)	0
Overweight (25≤BMI<30)	11 (28.2)	4 (80.0)
Obese (BMI≥30)	19 (48.7)	1 (20.0)
Race/ethnicity		
Non-Hispanic White	34 (87.2)	5 (100.0)
Non-Hispanic Black	1 (2.6)	
Hispanic	3 (7.7)	
Other	1 (2.6)	
Osteoarthritis family history	30 (76.9)	4 (80.0)
Polyarticular osteoarthritis	33 (84.6)	3 (60.0)
Current smoking	4.0 (10.3)	0.0 (100.0)
History of Coronary Artery Disease	1 (2.3)	0
History of Thyroid Disease	14 (31.8)	0
Diabetes Mellitus	8 (18.2)	0
NSAIDs	7 (18)	1 (20)
Osteoarthritis disease duration (years)	8.9±6.7	8.6±4.4
Leptin (ng/mL)	32.2±23.5	18.5±13.4
Adiponectin high MW (ug/mL)	4.8±2.9	2.5±2.2
Adiponectin mid MW (ug/mL)	1.4±0.5	1.0±0.2
Adiponectin total (ug/mL)	7.9±3.6	5.3±1.0
Resistin (ng/mL)	7.3±3.5	9.4±1.5
Hand x ray Kallman scores	43.3±17.4	46.2±10.8
Pain (VAS 0–100 mm)*	47.7±23.7	66.0±8.9
Leptin/Adiponectin total ratio (10^−3^)	4.9±3.7	3.6±2.7

Notes: BMI, body mass index. MW, molecular weight.

* Data summarized from participants who entered secondary analysis of adipokines and pain (35 women and 5 men).

Characteristics of the participants and adipokine serum concentrations are shown in Table 1. All participants met hand OA classification criteria except one female patient with early OA symptoms, who did not enter into the analysis. The final study cohort consisted of 39 women (mean age 63.5) and 5 men (mean age 72.6). The majority of the participants were white (89%). Eight patients took non-steroid-anti-inflammatories (NSAIDs) for hand OA pain as intermittent analgesics, which included celecoxib, naproxen, ibuprofen, and meloxicam. Mean (standard deviation) Kallman scores were 43.3(17.4) in females and 46.2(10.8) in males.

Table 2 provides the results of unadjusted and multivariable adjusted linear regression models analyzing the relationship between Kallman scores and adipokine serum concentrations. The unadjusted analyses demonstrated that adipokines were not related to Kallman scores (R^2^ ranged from 0.00–0.05 in unadjusted analysis, all p-values>0.22). In analyses adjusted for gender, history of CAD, thyroid disease, and DM, only age (p<0.01) and OA disease duration (p<0.03) were found to be significantly associated with Kallman scores.

**Table 2:** Unadjusted and Adjusted Analyses of Hand X-ray Kallman Score as a Function of Each Adipokine and Significant Determinants.

	Unadjusted Analysis				Multivariable Linear Regression Adjusted Analysis*			
	β	SE	P	R^2^	β	SE	P	R^2^
**Women Only**								
Leptin	0.15	0.12	0.21	0.05	0.07	0.11	0.50	0.39
Adiponectin HMW	1.01	0.96	0.30	0.03	0.14	0.86	0.87	0.38
Adiponectin MMW	3.20	5.21	0.54	0.01	−5.05	4.93	0.31	0.40
Adiponectin Total	0.83	0.78	0.30	0.03	−0.05	0.71	0.95	0.38
Resistin	−0.20	0.83	0.81	0.00	−0.06	0.73	0.93	0.38
Ratio Lep/Adip Total	0.33	0.76	0.66	0.01	0.75	0.65	0.26	0.40
Age	1.09	0.30	0.00	0.27	0.85	0.33	0.01¶	0.38
Disease Duration	1.16	0.38	0.00	0.20	0.73	0.40	0.08¶	0.38
Coronary.Art. Disease.	8.92	17.79	0.62	0.01	1.82	16.32	0.91	0.38
Thyroid Disease	5.65	0.34	0.34	0.02	4.35	5.08	0.40	0.38
Diabetes Mellitus	−6.36	6.91	0.36	0.02	−5.43	5.90	0.36	0.38
**Women and Men**								
Leptin	0.14	0.11	0.22	0.04	0.07	0.10	0.51	0.35
Adiponectin HMW	0.92	0.89	0.31	0.02	0.15	0.81	0.85	0.35
Adiponectin MMW	2.63	4.83	0.59	0.01	−3.84	4.45	0.39	0.36
Adiponectin Total	0.77	0.73	0.30	0.03	−0.03	0.67	0.97	0.34
Resistin	−0.15	0.77	0.85	0.00	−0.12	0.66	0.86	0.35
Ratio Lep/Adip Total	0.29	0.71	0.69	0.00	0.64	0.60	0.29	0.36
Age	0.95	0.26	0.00	0.24	0.84	0.27	0.00¶	0.34
Disease Duration	1.11	0.36	0.00	0.19	0.80	0.35	0.03¶	0.34
Gender	−2.89	8.01	0.72	0.00	4.53	7.12	0.53	0.34
Coronary Art. Disease	8.56	17.04	0.62	0.01	2.26	15.60	0.89	0.37
Thyroid Disease	4.83	5.42	0.38	0.02	4.30	4.90	0.39	0.37
Diabetes Mellitus	−6.58	6.53	0.32	0.02	−5.37	5.70	0.35	0.37

Notes: In adjusted analyses, we retained only gender, history of coronary artery disease, thyroid disease, and diabetes mellitus, and the statistically significant covariates (age, disease duration), selected during model building as defined by p<0.10. Unadjusted linear regression analysis was not performed in male participants due to the small sample size (N = 5). HMW, High Molecular Weight. MMW, Middle Molecular Weight. β refers to the beta coefficient estimated in linear regression models. SE, standard error.

¶ With adipokine serum concentrations not forced into the models, and adjusted for gender, history of coronary artery disease, thyroid disease, and diabetes mellitus, only age and osteoarthritis disease duration were associated with Kallman scores, while history of coronary artery disease, thyroid disease, diabetes mellitus, and gender were insiginificant, as defined by p-value>0.05.

Table 3 provides the results of the secondary analysis for hand pain. In multivariable analyses, leptin serum concentration, BMI, and history of coronary artery disease were associated with hand pain (R^2^ = 0.36, all p-values<0.05). Of these three, only BMI was positively associated. Of note, in the adjusted analyses, resistin, ratio of leptin/adiponectin, history of thyroid disease, and gender showed R^2^ = 0.31–0.36 and p-values of 0.05–0.20. Radiographic hand OA severity was also not associated with VAS pain score (R^2^ = 0.0018 in unadjusted analysis, all p-values = 0.87).

**Table 3:** Unadjusted and Adjusted Linear Regression Analyses of Pain as a Linear Function of Each Adipokine and Significant Determinants.

	Unadjusted Analyses				Multivariable Linear Regression Adjusted Analyses			
	β	SE	P	R^2^	β	SE	P	R^2^
**Women Only**								
Leptin	0.01	0.02	0.40	0.02	−0.05	0.03	0.06	0.30
Adiponectin HMW	−0.01	0.16	0.94	0.00	0.07	0.15	0.62	0.22
Adiponectin MMW	−0.07	0.83	0.93	0.00	0.22	0.85	0.79	0.22
Adiponectin Total	−0.02	0.13	0.90	0.00	0.04	0.12	0.74	0.22
Resistin	−0.12	0.11	0.29	0.03	−0.19	0.11	0.08	0.30
Ratio Lep/Adip Total	0.10	0.11	0.35	0.03	−0.20	0.16	0.23	0.25
Coronary Art. Disease	−3.89	2.34	0.11	0.08	−4.42	2.33	0.06	0.21
Thyroid Disease	0.50	0.82	0.55	0.01	0.78	0.79	0.33	0.21
Diabetes Mellitus	0.62	0.96	0.52	0.01	0.43	0.90	0.64	0.21
Body mass index	0.10	0.05	0.07	0.10	0.10	0.05	0.06	0.21
X-ray Scores	−0.01	0.02	0.81	0.00	N/A	N/A	N/A	N/A
**Women and Men**								
Leptin	0.01	0.02	0.59	0.01	−0.05	0.03	0.04¶	0.36
Adiponectin HMW	−0.09	0.14	0.55	0.01	0.07	0.14	0.62	0.28
Adiponectin MMW	−0.35	0.78	0.66	0.01	0.20	0.79	0.80	0.28
Adiponectin Total	−0.07	0.12	0.57	0.01	0.04	0.12	0.74	0.28
Resistin	−0.09	0.11	0.42	0.02	−0.20	0.10	0.05	0.35
Ratio Lep/Adip Total	0.08	0.10	0.44	0.02	−0.19	0.15	0.20	0.31
Body mass index	0.10	0.05	0.06	0.09	0.24	0.08	0.00¶	0.36
Coronary Art. Disease	−4.10	2.27	0.08	0.08	−5.68	2.18	0.01¶	0.36
Thyroid Disease	0.11	0.78	0.89	0.00	0.94	0.71	0.20	0.36
Diabetes Mellitus	0.31	0.92	0.74	0.00	0.71	0.82	0.40	0.36
Gender	−1.82	1.08	0.10	0.07	−1.60	1.06	0.14	0.36
X-ray scores	−0.00	0.02	0.87	0.00	N/A	N/A	N/A	N/A

Notes: In adjusted analyses, we retained only gender, history of coronary artery disease, thyroid disease, and diabetes mellitus, and the other significant covariates selected during model building as defined by p value<0.10. Unadjusted linear regression analysis was not performed in male participants due to the small sample size (N = 5). HMW, High Molecular Weight. MMW, Middle Molecular Weight. BMI, body mass index. VAS, visual-analog-scale. β refers to the beta coefficient estimated in linear regression models. SE, standard error.

¶ With adipokine serum concentrations not forced into the models, adjusted for gender, history of coronary artery disease, thyroid disease, and diabetes mellitus, we found leptin, BMI, history of coronary artery disease were associated with pain, while thyroid disease, diabetes mellitus, and gender were insiginificant, as defined by p-value>0.05.

## Discussion

Given that prior work suggested an association between adipokines and OA [3], [9], [10], we assessed the cross-sectional relationship between adipokines and Kallman hand x-ray scores. A secondary analysis examined adipokines' relationship with hand pain as literature suggested a link between them [11], [12]. Our study found leptin, BMI, and history of coronary artery disease were associated with chronic pain intensity of hand OA.

In a longitudinal study on radiographic hand OA progression and adipokines by Yusuf et al., radiographic joint space narrowing scores were analyzed, focusing on the cartilage component of joint changes [9]. Adiponectin was found to be associated with lower risk for hand OA radiographic progression [9]. In a cross-sectional study on adipokines and hand OA by Choe et al., using modified Kellgren-Lawrence scoring, radiographic changes in joints were classified into groups with or without radiographic hand OA [10]. Their analyses suggested cross-sectional association of serum resistin levels and radiographic hand OA [10]. However, our studies did not find evidence to support the association between adipokine serum concentrations and radiographic severity of hand OA. Literature also suggested an association between coronary artery disease and hand OA radiographic severity, but our study did not provide supporting evidence for this hypothesis [13], [14].

It is important to consider the strengths and limitations of our study. Our study used a newly recruited cohort. In addition, we used validated Kallman grading that offers a continuous numeric scoring and comprehensive surveillance of joint components including both cartilage and bone in most joints implicated in hand OA [5]–[7]. This further allowed linear regression modeling and afforded greater sensitivity to capture a relationship.

Several considerations limit the interpretation of our result. Our sample size was small, precluding our capacity to detect small but potentially clinically important effects. Blood samples were not collected simultaneously with the x-rays, in some cases 2–3 years apart. However, studies have suggested that the intra-individual adipokine system remains stable over time [15], [16]. Prior studies presented evidence to validate the utility of single adipokine measurements in population-based analyses [15], [16]. VAS pain scores were based on the assessments of the “typical pain score on an average day” and therefore represented chronic pain level in the past 2–3 years. The radiographic inter- and intra-reader correlations were moderate (0.71 and 0.78).

It is possible that adipokines and hand OA severity appear not associated because we missed a temporal window of association. From a biological standpoint, early hand OA can have distinct molecular-cellular signatures but can often lack sufficient evidence for clinical classification. The timing of adipokine involvement in OA development is also unclear. Should such association exist in early OA, ACR criteria would not allow the classification of such cases as OA. Our cross-sectional design would have limited ability to capture the temporal pattern of such an association.

Further, other factors may confound the relationship between hand OA radiographic severity and adipokines. Our multivariable regression model was developed using known and measurable potential confounders: age, gender, race, BMI, family history, polyarticular OA, and disease duration. Potential confounders such as genetic difference, hand anatomical variation, other co-morbidities, environmental factors, and occupational risk factors were not included in our analysis.

Lastly, we examined the relationship between adipokines and hand pain. Gandhi et al. showed that adiponectin-leptin ratio of knee synovial fluid was associated with the Short Form McGill Pain Questionnaire score [12]. Our results suggested that leptin serum concentration, BMI, and history of coronary arterty disease were associated with chronic pain perception of hand OA. History of coronary artery disease appeared to have a negative correlation with the intensity of hand OA pain. This has not been previously reported based on our literature search, although one should exercise caution in interpreting this result given the limitations of this study.

Resistin, ratio of leptin/adiponectin, thyroid disease, and gender had p values≤0.20, suggesting a potential association with OA pain. The small sample size may not have allowed the detection of these associations.

Similar to the apparent discordance between knee osteoarthritis radiographic severity and pain [17], our study suggests that osteoarthritis radiographic severity and pain may also be discordant in non-weight bearing joints. Although VAS pain scores measure only limited components of pain perception, its effectiveness has been validated and proved comparable to Western Ontario and McMaster Universities Arthritis Index (WOMAC) pain subscale [8].

## Conclusions

In conclusion, this study did not find supporting evidence for a cross-sectional association between adipokine serum concentrations and radiographic hand OA severity. The most important variables for joint damage were age and disease duration. In non-weight bearing joints, severity of joint damage was not associated with intensity of osteoarthritic pain. However, BMI, leptin serum concentration, and history of coronary artery disease were associated with chronic hand OA pain.
